# Variation in Primary Care Diagnostic Codes Used to Define Health Conditions for Research: Comparison of 45 Health Data Research UK Phenotype Library Codelists for Eight Health Conditions

**DOI:** 10.23889/ijpds.v11i1.3418

**Published:** 2026-09-24

**Authors:** Brian Kelly, Clare MacRae, Regina Prigge, Harry Hemingway, Natalie Fitzpatrick, Ana Torralbo, Bruce Guthrie

**Affiliations:** 1 Advanced Care Research Centre, Usher Institute, The University of Edinburgh, Usher Building, 5-7 Little France Road, Edinburgh EH8 9AG; 2 Institute of Health Informatics, University College London, 222 Euston Road, London, NW1 2DA

**Keywords:** Electronic Health Record, administrative data research, epidemiologic studies

## Abstract

**Introduction:**

The use of electronic healthcare records (EHRs) has become standard practice in most healthcare settings in many countries. These can be utilised for research purposes by developing codelists to define cohorts and health conditions of interest. In the UK the Health Data Research UK (HDR UK) Phenotype Library is a resource where researchers can access multiple codelists deposited by other researchers for a range of medical conditions.

**Objectives:**

This paper examines variation in the published codelists in the HDR UK Phenotype Library for a number of conditions.

**Methods:**

We extracted codelists held within the HDR UK Phenotype Library for eight common conditions (asthma, autism, coronary heart disease, chronic obstructive pulmonary disease, depression, hypertension, obesity, and type 2 diabetes). We categorised included codes as diagnostic, disease related, administrative, and unrelated to the condition. We explored the degree of agreement between codelists by estimating kappa statistics for codelists for each condition and category.

**Results:**

There were 48 published codelists for the eight conditions, with wide variation in the number of codes used to define each condition (for example from 39 to 247 codes in eight asthma codelists). Only codelists for chronic obstructive pulmonary disease and depression consistently achieved fair (kappa 0.21-0.40) moderate agreement (kappa 0.41-0.60), with other conditions consistently achieving fair agreement at best. Agreement was higher for diagnostic codes, and lowest for administrative codes.

**Conclusions:**

The HDR UK Phenotype Library publishes researchers’ codelists to facilitate greater transparency and replicability of studies. This study finds that agreement between codelists in terms of the codes included was low. Further research is needed to determine how such variation alters identification of which individuals have conditions of interest.

## Introduction

The use of electronic healthcare records (EHRs) has become standard practice in most healthcare settings in many countries [1, 2]. EHRs are a valuable resource for epidemiological research, as they are readily available to researchers and include rich longitudinal data on entire populations [3]. EHRs vary in how complete a record they are of an individual’s healthcare history, but primary care EHRs are often the most complete such record, particularly in countries like the UK with strong primary care systems where primary care acts as a gatekeeper to specialist services [4, 5]. This is particularly true in UK primary care, where all residents are registered with a single general practice and records follow the patient if they change practices. Primary care EHR data is typically a mix of free-text notes and coded data of various kinds including diagnoses, symptoms, administrative data, test results, and medications. UK primary care records became nearly fully computerised in the early 2000s [6]. Most UK primary care practices historically coded events and other information about patients using Read codes, a comprehensive computerised semi-hierarchical clinical classification system [7]. Although these are in the process of being replaced by the Systematised Nomenclature of Medicine Clinical Terms [8], many datasets including SAIL Databank remain coded in Read.

To utilise EHRs for research purposes, it is necessary to define cohorts of interest, for example by identifying patients with particular health conditions [9]. This involves creating lists of codes that indicate that the patient has the condition of interest. Codelists may include diagnostic codes, but can also include codes that refer to the use of medication (for example, medications used for exclusively for the management of dementia) and administrative codes (for example, attendance at a diabetes clinic can be used to define the presence of diabetes). However, some medications can be used for multiple diseases and some administrative codes do not mean the presences of a disease, for example referral for screening or a test. In some circumstances, the ability of codelists to identify patients with a health condition can be improved by combining multiple data elements, such as diagnostic codes, symptom codes, medications, and test results [10, 11]. Some codes may indicate inclusion in the condition of interest, while others indicate exclusion which requires consideration of the clinical term attached to codes rather than assuming that all codes in a particular part of the hierarchy indicate the presence of the condition.

However, there are significant challenges that researchers face when using EHRs to define health conditions of interest. Firstly, routine healthcare data is recorded to support medical care for the individual and for administrative purposes like payment, not for research purposes, and clinicians themselves do not use codelists during clinical care [9]. Historically, researchers have therefore created their own codelists, ideally in a structured process that involves clinicians. However, many research studies do not report details of codes and algorithms used to create research cohorts [12–14], reducing reproducibility [13]. Secondly, even if adequately reported, researchers with differing clinical or epidemiological experience often have different views of the inclusion of certain codes; and levels of agreement between codelists or between individual researchers can be low [12]. Often it is not possible to achieve consensus on many aspects of defining a condition or describing its sequelae [14]. This can often lead to large variations in the incidence and prevalence of conditions of interest, depending on the codelists and algorithms applied to the data [15]. Finally, the codes used by clinicians may change over time, either because new codes are created or because of changing clinical or administrative needs, so historical research codelists may also need updating [16, 17].

The choice of codelist is therefore very important for researchers, there is a risk of misclassification [18–20], which has implications when comparing results from different studies. These choices are not commonly reported, and there are calls to improve the reporting of choices made in developing codelists [9, 13, 21, 22]. In response, a number of resources have been created in recent years with the intention of documenting codelists used and phenotype definitions [23]. A UK example is the Health Data Research UK (HDR UK) Phenotype Library [24], a comprehensive, open access resource providing the research community with information, tools and phenotyping algorithms for UK electronic health records. By providing access to codelists and metadata deposited by researchers, the HDR UK Phenotype Library aims to support improvements in the quality of health data analysis through greater transparency [23]. Within the HDR UK Phenotype Library there are multiple codelists for any given condition. However, researchers still have to choose which to use, and it is unclear to what extent the available codelists vary.

This paper therefore examines how codelists for eight conditions published in the HDR UK Phenotype Library vary in terms of the Read codes included, including in how they vary by the types of code included, that relate to the same condition, for example, diagnostic, medication, procedure, and administrative codes?

## Methods

### Data

We extracted codelists held within the HDR UK Phenotype Library [24] for eight common conditions: asthma, autism, coronary heart disease (CHD), chronic obstructive pulmonary disease (COPD), depression, hypertension, obesity, and type 2 diabetes (accessed between 20^th^ January 2026 and 10^th^ March 2026, see supplementary material for exact dates). We focused on codelists that used Read codes, because UK primary care EHRs has more complete ascertainment of conditions than hospital data [4, 5].

### Data Management

We report the contents of each codelist held within the HDR UK Phenotype Library for each of the eight conditions of interest, excluding codelists with multiple records where the same author had uploaded identical codelists for the same condition. Since Read codes are used for a variety of purposes beyond diagnosis (for example, to record clinic attendance for administrative or payment reasons), we classified the Read codes in each codelist into categories in order to better understand if variation between codelists at least partly reflected broad choices about the type of codes to include as well as choices about each code individually. Supplementary material section 10 details the process used and provides examples, but in brief, our original intention was to distinguish diagnostic and administrative codes, but this was elaborated through discussion and consensus for codes which did not easily fit this dichotomy (for example, Read codes for the resolution of a diagnosis are evidence that the person had the condition at some point, but are not diagnostic on the date they are recorded). One author (BG) developed the initial set of categories which were then applied by BK, CM and RP and finalised in discussion. All four then classified all codes and the classification applied where there was universal agreement. If there was any disagreement, then BG and CM (both clinical academics who are also general practitioners and therefore users of the underlying electronic health records) reached a consensus on classification. The final classification included eight categories: diagnostic, administrative, diagnostic resolved, disease related event, disease related monitoring/ management, medication only for the condition, medication also for other conditions, and code unrelated to the condition. Diagnostic codes were those we considered to have no other function than to record a diagnosis. Disease related codes were those either for events which also named a diagnosis (for example, codes for emergency hospital admission for a named condition) or codes for condition monitoring or management which named a diagnosis (for example, medication review for a named condition). Administrative codes were those recording administrative events which named a condition (for example, recording attendance or non-attendance at a diabetes clinic). We considered some codes included in codelists as unrelated to the condition being defined; either completely unrelated (for example, ‘cardiac asthma’ is not asthma), or too non-specific to be certain (for example, ‘dietary advice for diabetes’ could be advice given to someone with pre-diabetes).

### Data Analysis

Initial descriptive analysis documented for each condition the number of codes used in each codelist, and the overlap between codelists. In order to explore the degree of agreement between codelists we estimated Cohen’s kappa statistics between all possible pairs of codelists within each condition with 95% confidence intervals calculated using bias-corrected bootstrapping using the kapci command in Stata BE v18.0. We then estimate the overall kappa by condition for all codelists related to that condition, and separately for each category of Read code (diagnostic, administrative etc.). Kappa can vary between −1 (complete disagreement) to 1 (complete agreement, with zero representing no agreement beyond chance. The kappa result be interpreted as follows: values ≤ 0 as indicating worse than chance agreement and 0.01–0.20 as none to slight, 0.21–0.40 as fair, 0.41– 0.60 as moderate, 0.61–0.80 as substantial, and 0.81–1.00 as almost perfect agreement [25]. We use summary tables and figures to illustrate these results, and report the full findings in supplementary material. Since analysis only uses published codelists, there was no requirement for ethical review.

### Results

Forty-two codelists were identified for the eight included conditions. Within each condition, there was wide variation in the number of codes used to define that condition (Table 1, supplementary tables S1-S8). Asthma had eight codelists, containing between 39 and 247 codes (334 distinct codes in total). Autism had three codelists, containing between 8 and 19 codes (21 distinct codes). CHD had eight codelists, containing between 30 and 129 codes (130 distinct codes). COPD had five codelists, containing between 41 and 136 codes (136 distinct codes). Depression had nine codelists, containing between 88 and 135 codes (187 distinct codes). Hypertension had eight codelists, containing between 19 and 130 codes (145 distinct codes). Obesity had four codelists, containing between 21 and 70 codes (90 distinct codes). Type 2 diabetes had three codelists, containing between 102 and 645 codes (646 distinct codes).

**Table 1:** Number of Read Codes in Each Codelist examined, and How Often Codes in One Codelist Were Also Included in All Codelists for Each Condition

Codelist^*^	**No. of codes included in codelist**	**% of codes only in this codelist**	**% in one or more other codelists**	**% in all codelists for the condition**
Asthma 1	247	33.6	55.4	10.9
Asthma 2	165	9.1	74.5	16.4
Asthma 3	156	34.0	48.7	17.3
Asthma 4	146	0	81.5	18.5
Asthma 5	42	0	35.7	64.3
Asthma 6	40	0	32.5	67.5
Asthma 7	40	0	32.5	67.5
Asthma 8	39	0	30.8	69.2
Autism 1	19	21.1	42.1	36.8
Autism 2	16	6.3	50.0	43.8
Autism 3	8	12.5	0	87.5
CHD 1	129	0	81.5	18.6
CHD 2	129	0	81.5	18.6
CHD 3	62	0	61.3	38.7
CHD 4	62	0	61.3	38.7
CHD 5	30	3.3	16.7	80.0
COPD 1	136	35.3	46.3	18.4
COPD 2	67	0	62.7	37.3
COPD 3	58	0	56.9	43.1
COPD 4	55	0	54.5	45.5
COPD 5	41	0	39.0	61.0
Depression 1	135	11.9	42.9	45.2
Depression 2	131	22.9	30.5	46.6
Depression 3	121	4.1	45.6	50.4
Depression 4	107	0	43.0	57.0
Depression 5	105	0	42.0	58.1
Depression 6	105	0	42.0	58.1
Depression 7	100	0	39.0	61.0
Depression 8	100	0	39.0	61.0
Depression 9	88	0	30.6	69.3
Hypertension 1	130	15.4	84.6	0
Hypertension 2	85	0	100	0
Hypertension 3	83	0	100	0
Hypertension 4	76	1.3	98.7	0
Hypertension 5	69	0	100	0
Hypertension 6	65	0	100	0
Hypertension 7	61	3.2	96.7	0
Hypertension 8	19	0	100	0
Obesity 1	70	38.6	40.0	21.4
Obesity 2	52	23.1	48.1	28.8
Obesity 3	28	14.3	32.1	53.6
Obesity 4	21	19.0	9.6	71.4
Type 2 diabetes 1	645	82.9	2.2	14.9
Type 2 diabetes 2	105	1.0	7.6	91.4
Type 2 diabetes 3	102	0	5.9	94.1

^*^Supplementary tables S1a-c (asthma), S2a-c (autism), S3a-c (CHD), S4a-c (COPD), S5a-c (depression), S6a-c (hypertension), S7a-c (obesity) and S8a-c (type 2 diabetes) document all 42 codelists examined.

In general, codelists which included more codes had a higher percentage of codes which were unique to that codelist and had a lower percentage of codes which were present in all codelists for the same condition, but there is variation between the health conditions (Table 1). For example, for the eight hypertension codelists examined, no single code was present in all eight codelists, but with the exception of the hypertension 1 codelist (Mansfield et al 2021) almost all codes were included in more than one codelist. For the nine depression codelists, most codes included in individual codelists were included in all nine codelists (ranging from 45% to 58%). In contrast, for asthma there was much more variation in the percentage of codes included in individual codelists which were included in all eight asthma codelists (ranging from 11% to 69%).

Figure 1 illustrates the proportion of codes for each condition in each category of Read code. Overall, 67% of codes examined were classified as diagnostic, 15% were disease related, 8% were administrative, and 10% were unrelated to the condition. This varied by condition; for example, 87% of Read codes for CHD were classified as diagnostic compared to 26% for asthma, and 34% of Read codes for COPD were classified as unrelated to the condition compared to 12% for hypertension. Overall, diagnostic codes were most frequent in CHD and autism, and relatively high in depression, hypertension, and type 2 diabetes; and least frequent in asthma. Disease related codes were most frequent asthma and obesity and least frequent in depression and autism. Administrative codes were most frequent in type 2 diabetes, and relatively high in obesity and COPD; and least frequent (absent) in autism. Codes that were unrelated to the condition were most frequent in COPD, depression, and asthma; and least frequent in CHD.

**Figure 1:**
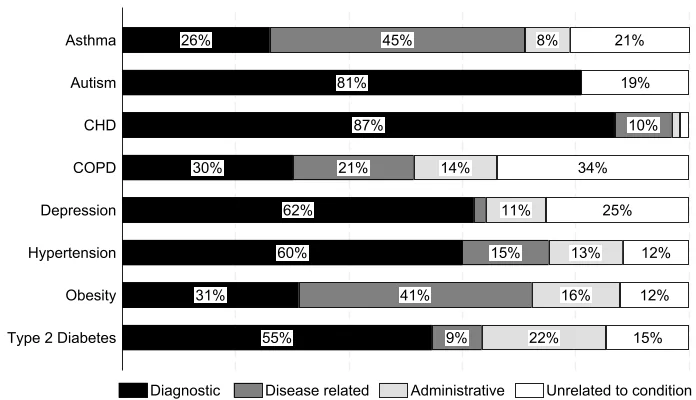
Classification of Unique Read Codes by Condition^*^

Figure 2 shows pairwise kappa statistics for each codelist and all other codelists for the same condition (full data is shown in supplementary tables S11a-S11h). There is variation in the level of agreement between pairs of codelists within each condition. There is no to, at best, fair agreement for asthma, autism, hypertension, and obesity; fair agreement for CHD; moderate agreement for COPD and depression. Agreement for diabetes is zero for one codelist, and moderate for the other two.

**Figure 2:**
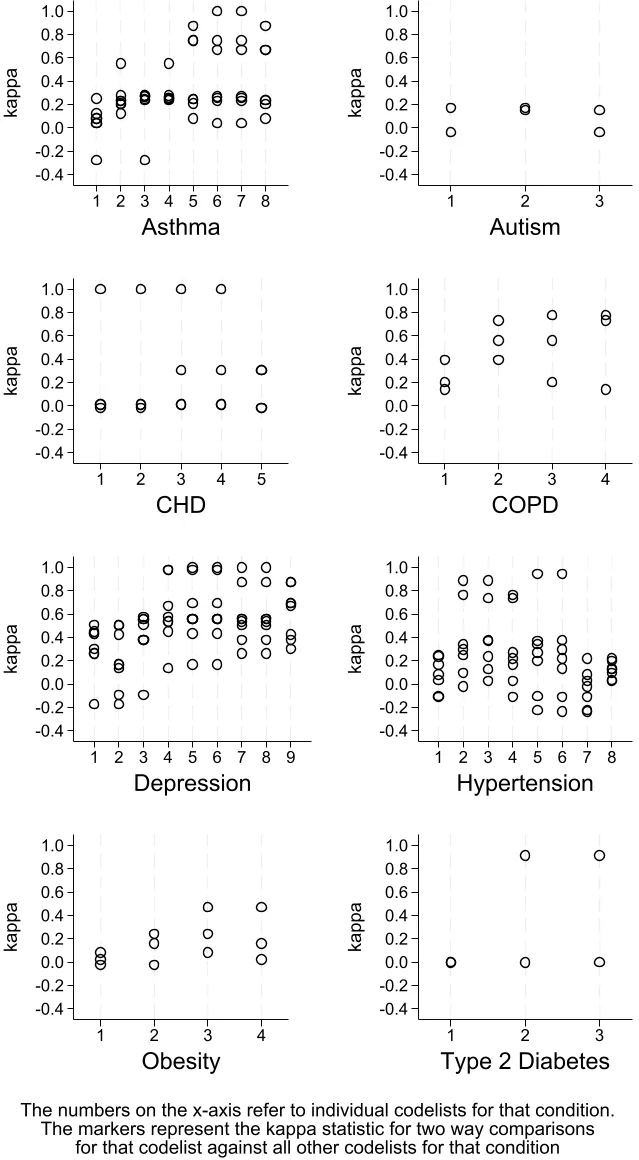
Pairwise Kappa Statistics Comparing Agreement Between All Pairs of Codelists for Each Condition

Kappa values can be interpreted as follows: values ≤0 as indicating worse than chance agreement and 0.01–0.20 as none to slight, 0.21–0.40 as fair, 0.41– 0.60 as moderate, 0.61–0.80 as substantial, and 0.81–1.00 as almost perfect agreement.

Figure 3 shows the overall kappa statistic by condition of interest, broken down by classification of Read code (the underlying kappa results are in supplementary material tables S12a and S12b.) Overall, there are low levels of agreement between all types of Read codes in codelists. Again, this varies by condition, being lowest for type 2 diabetes and highest for depression. Agreement tends to be highest for diagnostic Read codes (with the exception of autism), and lowest for administrative Read codes (which have mostly negative kappa).

**Figure 3:**
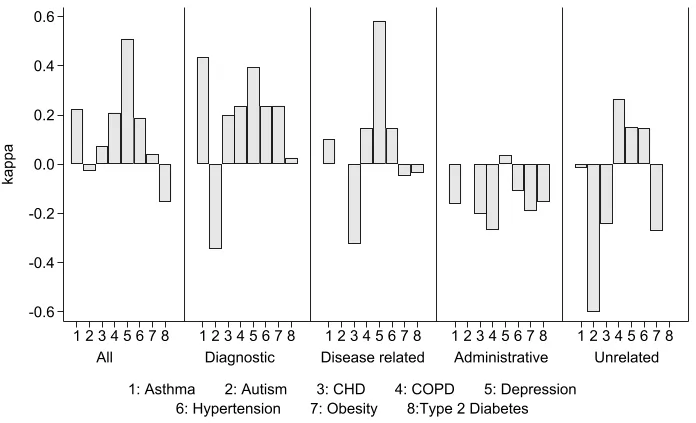
Overall Kappa Statistic by Condition of Interest, Broken Down by Classification of Read Code^*^

^*^Because of small numbers of codes, diagnostic in figure combines: (a) diagnostic, (b) medication only for the condition; Disease related combines: (a) disease related event, (b) disease related monitoring/management; Unrelated to condition combines: (a) unrelated to condition, (b) medication also for other conditions.

Kappa values can be interpreted as follows: values ≤0 as indicating worse than chance agreement and 0.01–0.20 as none to slight, 0.21–0.40 as fair, 0.41– 0.60 as moderate, 0.61–0.80 as substantial, and 0.81–1.00 as almost perfect agreement.

## Discussion

### Summary of Findings

This paper aimed to explore variation between 45 HDR UK Phenotype Library codelists for eight health conditions, and the extent to which this variation differed by the classification of Read code (diagnostic, disease related, administrative, and codes unrelated to the condition). We found there was considerable variation in terms of the Read codes used to define the eight conditions we examined. For example, there are eight codelists for asthma containing between 39 and 247 Read codes. Codelists varied in the extent to which they included different categories of Read code. We found that there were generally low levels of agreement between codelists for the same condition; though this varied by condition. Codelists for depression had the greatest level of agreement (kappa=0.510 consistent with moderate agreement), but all other conditions had a kappa lower than 0.250 (consistent with poor or minimal agreement), and both autism and type 2 diabetes had a kappa <0 (agreement less than would be expected at random). Not unexpectedly, all codelists included a high proportion of diagnostic codes, with more variation in whether they included other types of codes. For codelists using particular categories of codes, we found levels of agreement between codelists was greater for diagnostic and disease related codes, and lowest for administrative and unrelated codes.

### Strengths and Limitations

The main strength of this paper is that it systematically examines the extent to which published codelists agree with each other, which is under-researched. A further strength is that we used well documented primary care codelists from a single phenotype library in the UK, although this does mean that it is uncertain if the findings apply in other contexts: however, we think it very likely that this is a general problem. There are a number of limitations. First, codelists may legitimately differ because researchers have different aims or uses for the codelist, although this is not explicit in the phenotype library metadata. Related to this, our classification of Read codes into categories was arbitrary (although carried out in a systematic way), but this reflects the kinds of choices which researchers have to make when choosing codes. Second, since new Read codes were added over time and coding practices varied over time, observed differences in codelists could reflect the different set of codes available or used when the codelist was created.

### Comparison with Other Literature

Our findings are consistent with previous research that identified variations in codelists used to identify health conditions [14, 15], and found that levels of agreement between different codelists can be low [12, 13]. This paper extends previous work by examining a much wider range of conditions and codelists, and by looking at agreement between different classifications of Read codes. Variation is likely to be less in other coding systems like ICD-10 because they have fewer codes and are less granular, but conversely may be worse in the future in coding systems containing many more codes than Read such as SNOMED-CT.

### Implications for Researchers

Our findings that codelists vary considerably in their choice of codes has a number of implications. Publication of codelists in phenotype libraries has improved the transparency and replicability of research, but the existence of multiple codelists with limited validation (or limited validation in the dataset being used) presents researchers with difficult choices in the face of multiple codelists on offer. Researchers could use different sets of codelists and conduct sensitivity analysis [12]. However, this is resource intensive and may not provide consistency required to deal with problems of different results arising from different codelists. There have also been calls to harmonise and standardise codelists for conditions of interest, which could lead to a single consensus codelist, or to a minimum set of codes for each condition of interest [26], but it is also important to recognise different purposes may require the use of different codes meaning that there will not always be a ‘gold standard’ set of codes and associated algorithms to define conditions [12]. Even if there were a ‘gold standard,’ it could be subject to drift over time because condition classifications and choice of codes by clinicians may change over time [16], for example when codes are used for payment [27].

Guidance on which codelists to employ for research is outside the current aims of the HDR UK Phenotype Library. Rather, the stated aim of the HDR UK Phenotype Library is to provide a place for researchers to publish findable, consistently documented codelists, rather than to standardise or harmonise disease definitions [28] Although the HDR UK Phenotype Library intends to better document how codelists have been validated [28], the onus remains on individual researchers to choose which phenotypes or codelists they wish to use. Similar issues apply to other phenotype libraries [29], and other coding systems, with CIPHER for example offering ICD-10 codelists which vary in terms of included codes (see for example, codelists for autism https://phenomics.va.ornl.gov/web/cipher/search?search= autism).

In practice therefore, researchers need to carefully consider the accuracy of published codelists (including evidence of their validity), and their relevance to the researcher’s purpose and dataset (including evidence of their validity in a particular context [30, 31], since clinicians in different contexts vary in how they use clinical codes [17, 32–34]). The aim has to be to minimise measurement error which can lead to observing smaller effect sizes than exist if conditions were accurately defined [35]. Ideally, researchers would either use codelists or algorithms validated in the data they are using, although this may not be feasible requiring judgements whose justification should be documented in publications.

There have been suggestions that that the answer to all the above problems is that healthcare systems should be more standardised in how they use codes [27]. The UK Quality Outcomes Framework (QOF) is an example of such standardisation for conditions included in primary care pay-for-performance [36, 37]. The introduction of QOF in the UK led to improved recording of conditions [17]. However, such standardisation is always likely to be local, meaning that researchers still have to choose the best codelist or algorithm for their purpose and their context.

### Future Research

There is a need for further research to determine how much difference variation between codelists makes to the individuals identified as having different conditions, and how the use of different categories of codes (such as administrative codes) alters the performance of codelists in identifying individuals with specific conditions.

## Conclusion

Phenotype libraries help improve the transparency and reproducibility of research by publishing the codelists and algorithms used by researchers. However, published codelists vary considerably, meaning that researchers still need to think carefully about which codelist they apply in terms of its validity in their data for their particular purposes.

## Data Availability

All codelists used in the analysis are freely available in the HDR UK Phenotype Library: https://phenotypes.healthdatagateway.org/.

## Abbreviations

EHRs:	Electronic Healthcare Records
ICD:	International Classification of Diseases
HDR UK:	Health Data Research UK
NHS:	National Health Service (UK)
SNOMED CT:	Systematised Nomenclature of Medicine Clinical Terms
